# DNA methylation promotes paired box 2 expression via myeloid zinc finger 1 in endometrial cancer

**DOI:** 10.18632/oncotarget.12626

**Published:** 2016-10-13

**Authors:** Nan Jia, Jieyu Wang, Qing Li, Xiang Tao, Kaikai Chang, Keqin Hua, Yinhua Yu, Kwong-Kwok Wong, Weiwei Feng

**Affiliations:** ^1^ Department of Gynecology, Obstetrics and Gynecology Hospital, Fudan University, Shanghai, China; ^2^ Department of Pathology, Obstetrics and Gynecology Hospital, Fudan University, Shanghai, China; ^3^ Shanghai Key Laboratory of Female Reproductive Endocrine-Related Disease, Fudan University, Shanghai, China; ^4^ Department of Gynecologic Oncology and Reproductive Medicine, The University of Texas MD Anderson Cancer Center, Houston, TX, USA

**Keywords:** paired box 2, myeloid zinc finger 1, endometrial cancer, MassARRAY, DNA methylation

## Abstract

This work investigated the role of paired box 2 (PAX2) in endometrial cancer and its epigenetic regulation mechanism. Endometrial cancer tissues and cell lines exhibited increased PAX2 expression compared with hyperplasia, normal endometrium and endometrial epithelial cells. Knock-down of PAX2 resulted in reduced cell viability, invasion and migration, and PAX2 overexpression caused the opposite effects. Increased methylation of the PAX2 promoter was observed in both cancer tissues and cell lines and was positively correlated with PAX2 expression. After 5-Aza-CdR treatment, PAX2 mRNA and protein were down-regulated, and PAX2 methylation was decreased. Deletion analysis confirmed that a repressive transcriptional regulatory region of the PAX2 promoter coincided with the hypermethylated region identified in MassARRAY analysis. Binding sites of myeloid zinc finger 1 (MZF1) are predicted in the defined region. Knock-down of MZF1 up-regulated the transcriptional activity and protein level of PAX2 after 5-Aza-CdR treatment, which indicated that MZF1 may act as a repressive transcription factor when the PAX2 promoter is unmethylated. In conclusion, PAX2 is involved in the carcinogenesis of endometrial cancer by stimulating cell growth and promoting cell motility. The overexpression of PAX2 in endometrial cancer is regulated by promoter hypermethylation and the transcription factor MZF1.

## INTRODUCTION

Endometrial cancer (EnCa) is one of the most common cancers of women and has an increasing incidence worldwide. In addition to genetic changes, the dysregulation of genes by DNA methylation plays a significant role in tumor initiation and progression. However, the pathogenesis and progression of endometrial cancer related to gene dysfunction via epigenetic regulation are rarely reported.

Paired box 2 (PAX2) is a member of paired box family and encodes a DNA-binding protein that binds to the paired box domain-specific motifs. PAX2 is a major transcription factor involved in the development of urogenital system [1]. In recent years, research on PAX2 has gradually expanded to cancer, including kidney cancer [2], breast cancer [3], colon cancer [4], and cancers of the female reproductive tract. PAX2 is highly expressed in multiple tumors and is essential for tumor cell survival [5]. PAX2 is also a biomarker of mesonephric duct-derived tumors [6], and positive expression has been reported in 67% of papillary serous ovarian cancers [7] and low-grade ovarian cancer [8, 9]. PAX2 can function as a cancer promoter or suppressor depending on the genetic background [10]. Abnormal expression of PAX2 also occurs in endometrial cancer [11-20].

As an oncogene involved in the development of endometrial cancer, PAX2 is activated in the development of tamoxifen-induced endometrial cancer, which is related to hypomethylation-induced activation [11]. PAX2 is a downstream gene in the steroid hormone receptor signal pathway and is overexpressed in endometrial cancer and benign endometrial hyperplasia compared with normal controls [12]. PAX2 expression was also found to increase as the pathological malignancy progressed [13]. In a xenotransplanted tumor model of human endometrial cancer in nude mice, increased PAX2 expression was observed in tumors with poor cell differentiation, and tumor volume as well as expression of PCNA and Bcl-2 were decreased following knockdown of PAX2 [14].

However, other articles have reported that PAX2 expression is decreased in endometrial cancer. PAX2 was found to be constantly expressed in most epithelial cells of non-neoplastic tissues of Müllerian origin but less frequently observed in several types of endometrial cancer [15, 16]. PAX2 expression was reportedly lost in 73.3% of G1 endometrioid endometrial cancers [17] and in 77% of endometrial cancers, and this pattern correlated with pathological malignancy and PTEN deletion [18]. Loss of PAX2 and PTEN expression was also observed in an estrogen-induced endometrial hyperplasia mouse model [19], and clearance of PAX2-/PTEN-null glands occurred in response to therapy in endometrial hyperplasia tissues [20].

PAX2 is controlled by epigenetic regulation. The histone methylation and DNA remodeling of PAX2 play a significant role in the development of kidney [21]. In renal malignancies, the overexpression of PAX2 is regulated by DNA hypomethylation [22], which also occurs in tamoxifen-induced endometrial cancer [11] and endometriosis [23]. In mouse embryonic fibroblasts, menin up-regulates Wilms tumor gene 1 to recruit the polycomb-group protein complex and DNA-methyltransferase 1 to the PAX2 promoter and represses PAX2 expression via H3k27me3 and DNA hypermethylation [24].

Regardless, the function of PAX2 in endometrial cancer has not been clarified to date. Most studies have focused on the phenomenon of abnormal PAX2 expression, and the results are incomplete. Indeed, the mechanism of abnormal PAX2 expression in endometrial cancer, which we address in this study, is rarely reported.

## RESULTS

### PAX2 is overexpressed in epithelium of endometrial cancers

Immunohistochemistry was performed in 111 endometrium tissues, including 25 normal endometrium (20 proliferative and 5 atrophic), 23 endometrial hyperplasia (18 simple, 1 complex and 4 atypical), and 63 endometrial cancers (49 type 1 and 14 type 2).

**Figure 1:**
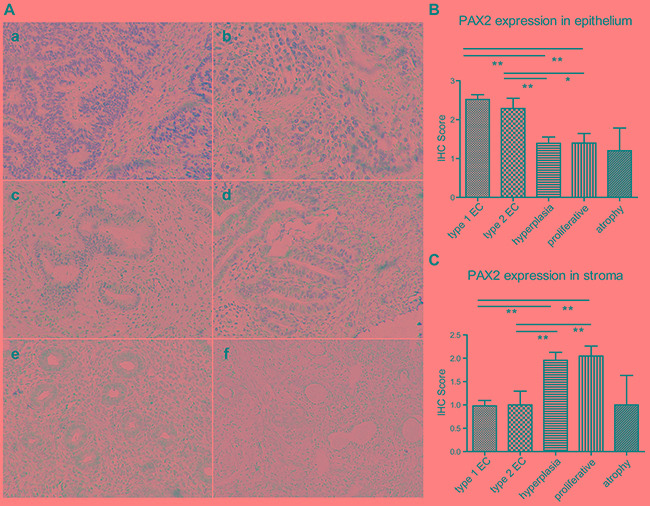
PAX2 expression in endometrial tissues **A.** PAX2 expression was increased in the epithelium of endometrial cancers compared with endometrial hyperplasia and normal tissues. a. type 1 endometrial cancer, b. type 2 endometrial cancer, c. complex hyperplasia, d. atypical hyperplasia, e. proliferative endometrium, f. atrophic endometrium. All images were obtained at 200x magnification using light microscopy. **B.** PAX2 expression in the epithelium was semi-quantified according to IHC results and compared between different groups. **C.** PAX2 expression in the stroma was semi-quantified according to IHC results and compared between different groups. **p<0.01, *p<0.05

PAX2 expression was increased in the epithelium of endometrial cancers compared with hyperplasia and normal endometrium. No difference was observed between type 1 and type 2 cancers or between hyperplasia and normal endometrium (Figure 1A and 1B). Interestingly, the stroma in the cancers exhibited reduced PAX2 expression compared with hyperplasia and proliferative endometrium; however, atrophic endometrium also exhibited reduced stromal PAX2 expression (Figure 1C).

### PAX2 stimulates cell growth and promotes motility of endometrial cancer cells

**Figure 2:**
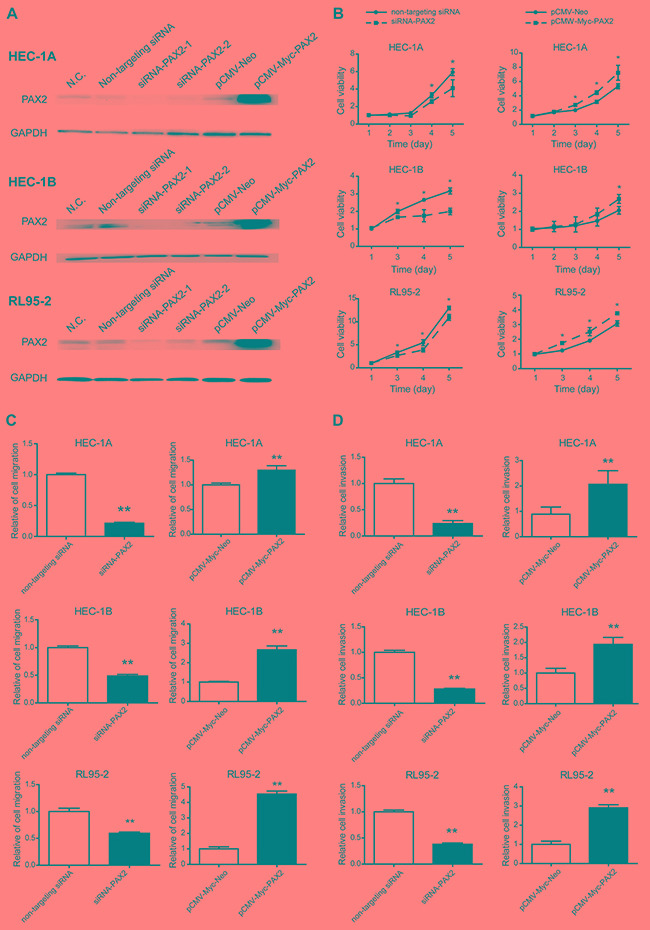
Knocking down or overexpressing PAX2 inhibited or stimulated, respectively, cell growth, migration and invasion *in vitro* **A.** PAX2 was knocked down or overexpressed by transfection of siRNA or pCMV-Myc-PAX2, respectively, in HEC-1A, HEC-1B and RL95-2 cells. **B.** Cell proliferation was reduced by knocking down PAX2 and stimulated by overexpressing PAX2 in HEC-1A, HEC-1B and RL95-2 cells. **C.** Cell migration was decreased in cells transfected with siRNA-PAX2 and increased in cells transfected with pCMV-Myc-PAX2. D. Cell invasion was inhibited in cells transfected with siRNA-PAX2 and stimulated in cells transfected with pCMV-Myc-PAX2. *p<0.05, **p<0.01.

Because PAX2 expression was increased in endometrial cancer tissues compared with normal endometrium, we assumed that PAX2 played an oncogenic role in the carcinogenesis of endometrial cancer. To verify our hypothesis, we first used siRNA or PAX2 cDNA transfection to transiently knock down or overexpress PAX2 and investigated cell viability. PAX2 siRNA treatment reduced PAX2 expression by 80%, and PAX2 cDNA transfection increased PAX2 expression by 2- to 3-fold (Figure 2A). Knocking down PAX2 reduced cell proliferation in a time-dependent manner in HEC-1A, HEC-1B and RL95-2 cells. The effect was noted at 48 or 72 hours and peaked at 120 hours. Consistent with this observation, PAX2 overexpression stimulated cell growth in all three cell lines (Figure 2B).

Cell migration was decreased in cells transfected with siRNA-PAX2 and increased in cells transfected with pCMV-Myc-PAX2 (Figure 2C). Cell invasion was weakened after transfection with siRNA-PAX2 and promoted after PAX2 overexpression (Figure 2D).

### PAX2 promoter is hypermethylated in endometrial cancer cells lines and tissues

**Figure 3:**
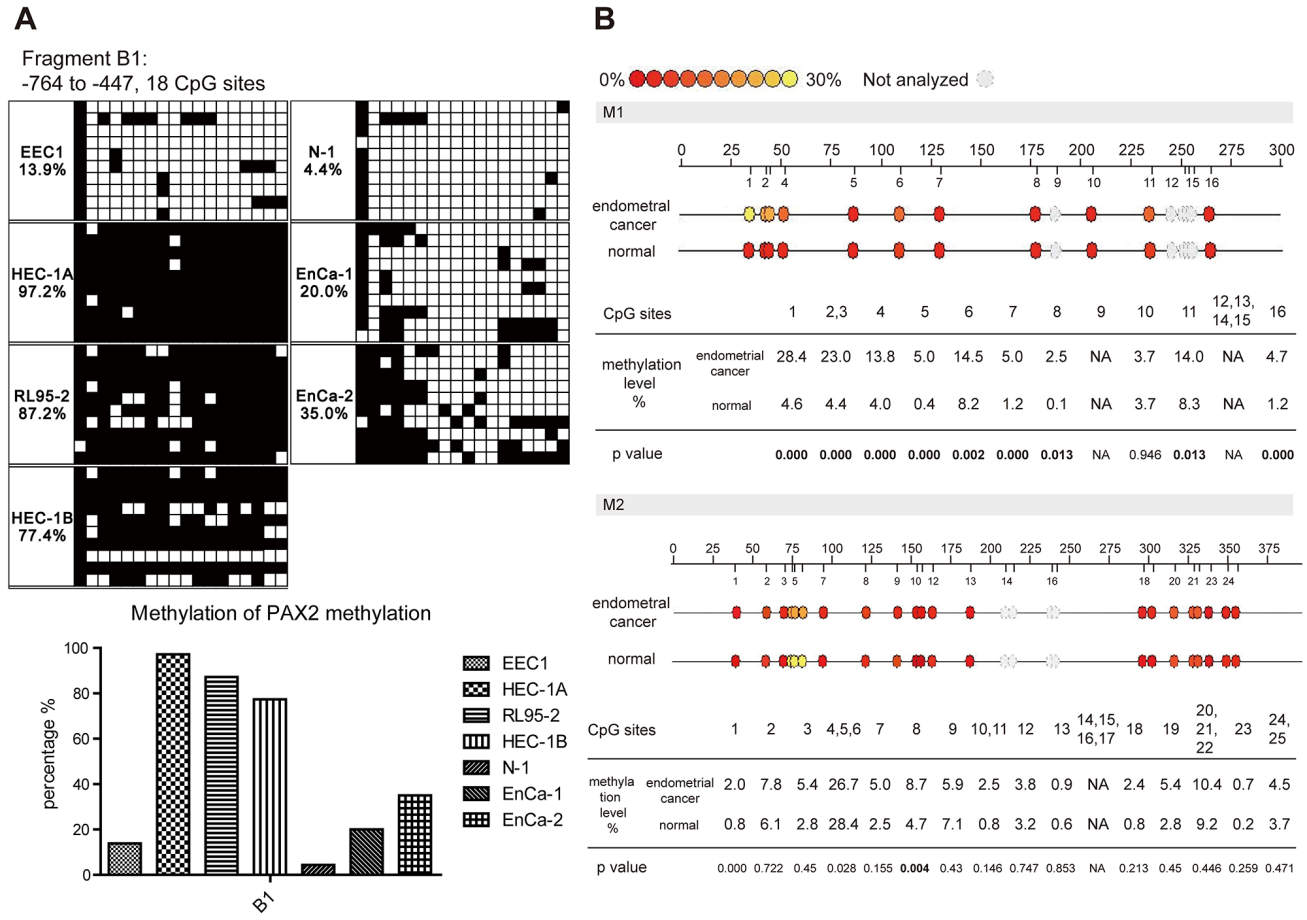
PAX2 promoter was hypermethylated in endometrial cancer cell lines and tissues **A.** Bisulfite sequencing results showed fragment B1 (-764 to -447 bp) of the PAX2 promoter was hypermethylated in endometrial cancer cell lines and tissues: rows represent clones (10 for each sample), columns represent CpG sites. Black squares represent methylated CpGs, and white squares represent unmethylated CpGs. EEC: endometrial epithelial cell. EnCa: endometrial cancer. N: normal. **B.** MassARRAY results indicate that PAX2 was hypermethylated in endometrial cancer tissues compared with normal endometrial tissues. M1 is an amplicon of a 280-bp fragment from -723 bp to -443 bp; M2 is an amplicon of a 379-bp fragment from -468 bp to -89 bp. Hypermethylated CpG sites were centralized in the 5’ of M1 (CpG 1 to 7).

To elucidate whether the expression of PAX2 is regulated by DNA methylation, we first investigated the methylation status of the PAX2 promoter in endometrial epithelial cells (EECs) and endometrial cancer cell lines and in 3 endometrial tissue samples by bisulfite sequencing. Increased methylation levels of the PAX2 promoter were observed in endometrial cancer cell lines. Fragment B1 amplifies the -767 to -447 region (318 bp) containing 18 CpG dinucleotide sites. As shown in Figure 3A and 3B, 13.9% of the CpG sites were methylated in EEC1, whereas 97.2%, 87.2% and 77.4% were methylated in HEC-1A, RL95-2 and HEC-1B cells, respectively. We also verified differential methylation in two tissue samples of endometrial cancer (EnCa) and 1 normal endometrium tissue sample (N) (EnCa-1 and EnCa-2 were 20.0% and 35.0% methylated, respectively; N-1 was 4.4% methylated).

Furthermore, we evaluated the methylation state of PAX2 in 99 endometrial tissues, including 59 type 1, 18 type 2 EnCa tissues, 20 proliferative and 2 atrophic endometrium tissues. The methylation level of -723 bp to -89 bp upstream of PAX2 promoter was assessed using the MassARRAY system. This region was divided into two fragments: M1 (-723 bp to -443 bp, containing 16 CpG sites) and M2 (-468 bp to -89 bp, containing 25 CpG sites) (Figure 3C). We compared the MassARRAY and bisulfite sequencing results in three samples and found that the methylation levels measured by the two methods were comparable (EnCa-1: 23.7% VS. 20.0%; EnCa-2: 31.2% VS. 35.0%; N-1: 4.4% VS. 1.9%), which indicated that the MassARRAY results were reliable. When each single CpG site was compared between cancer and normal tissues, the methylation levels of CpG 1-7 and CpG 11 of M1 were significantly increased in cancer patients. Methylation levels below 5% were considered noise. However, no differences in the methylation levels of CpG sites were noted between cancer and normal tissues. We then selected the region of CpG1-7 of M1 and used the average methylation level of the seven CpG sites as the methylation value of a sample. Using the average methylation level of CpG 1-7 of M1 plus two times the standard deviation of the pooled normal samples as a cut-off point (5.7%), there is a > 91% probability that the methylation level for a normal tissue will be lower than the cut-off point. It is reasonable to assume that a value larger than the cut-off point is likely to be abnormal (or hypermethylated). According to this assumption, 69.6% of the EnCa tissues and only 9% of the normal tissues were hypermethylated.

### PAX2 overexpression correlates with promoter hypermethylation, demethylating PAX2 down-regulates PAX2 expression

**Figure 4:**
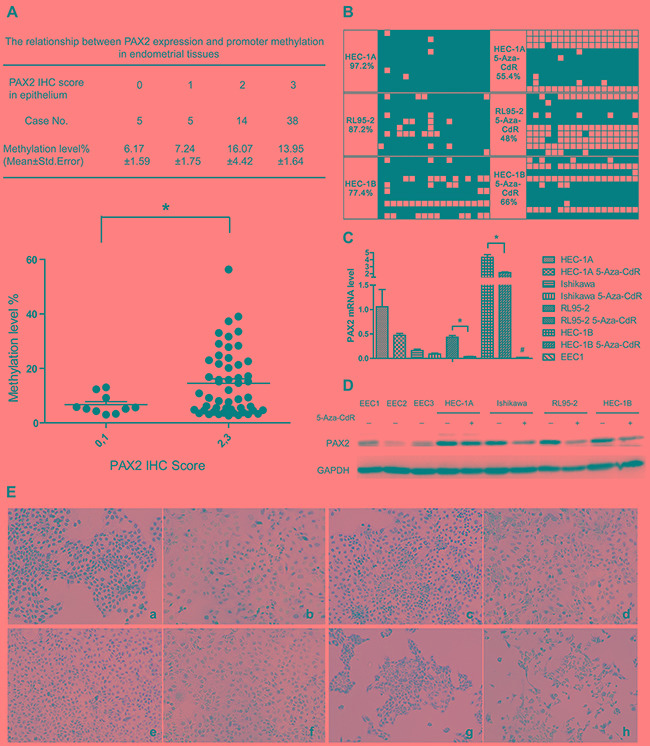
Increased methylation is correlated with PAX2 overexpression in endometrial cancer tissues, and PAX2 expression was down-regulated after demethylated by 5-Aza-CdR treatment **A.** PAX2 weak expression group (IHC score=0 or 1) exhibited reduced methylation levels compared with the strong expression group (IHC score=2 or 3). **B.** PAX2 promoter (fragment B1) of endometrial cancer cell lines was demethylated by 5-Aza-CdR treatment. **C.** PAX2 was down-regulated on mRNA level after 5-Aza-CdR treatment in RL95-2 and HEC-1B cells. *p<0.05. PAX2 expression in EEC1 was reduced compared with endometrial cancer cells. **D.** PAX2 protein expression was down-regulated after 5-Aza-CdR treatment in Ishikawa, RL95-2 and HEC-1B cells as measured by western blot. **E.** PAX2 protein was down-regulated after 5-Aza-CdR treatment in endometrial cancer cells measured by immunocytochemistry: a. HEC-1A control, b. HEC-1A 5-Aza-CdR, c. Ishikawa control, d. Ishikawa 5-Aza-CdR, e. RL95-2 control, f. RL95-2 5-Aza-CdR, g. HEC-1B control, h. HEC-1B 5-Aza-CdR. *p<0.05, # p<0.01.

Contrary to the normal pattern of hypomethylation-activated overexpression, PAX2 overexpression correlated with promoter hypermethylation in endometrial tissues. Specifically, the methylation level of CpG 1-7 of fragment M1 of PAX2 increased with its expression based on the immunohistochemistry (IHC) score (Figure 4A).

MassARRAY analysis indicated that the PAX2 promoter was hypermethylated in endometrial cancer tissues and cell lines. To determine whether PAX2 promoter methylation regulates gene expression, endometrial cancer cell lines were treated with the demethylating agent 5-aza-2’-deoxy-cytidine (5-Aza-CdR, decitabine) for 4 days (Figure 4B). After treatment, the methylation level of the PAX2 promoter decreased in endometrial cancer cell lines (HEC-1A: from 94.6% to 55.4%, RL95-2: from 59% to 48%, HEC-1B: from 96% to 66%. We did not measure the methylation of Ishikawa because the basic methylation level is 0.) PAX2 expression was also down-regulated at the mRNA and protein levels, as verified by real-time PCR (Figure 4C), western blot (Figure 4D) and immunocytochemistry (Figure 4E). PAX2 was overexpressed in endometrial cancer cells compared with primary cultured EECs, which was consistent with the immunohistochemistry results. Surprisingly, Ishikawa cells were hypomethylated before treatment with 5-Aza-CdR; however, PAX2 was also down-regulated by 5-Aza-CdR, as assessed by qPCR and western blot.

### Location of transcriptionally active region of the PAX2 promoter

The transcription start site (TSS) was confirmed by the Ensembl website. Deletion analysis of -900 bp to +100 bp of the PAX2 promoter was performed. Four fragments named P1 to P4 were amplified, and 4 luciferase reporter vectors (pGL3-P1, pGL3-P2, pGL3-P3 and pGL3-P4) were constructed by integration into the pGL3-basic vector.

**Figure 5:**
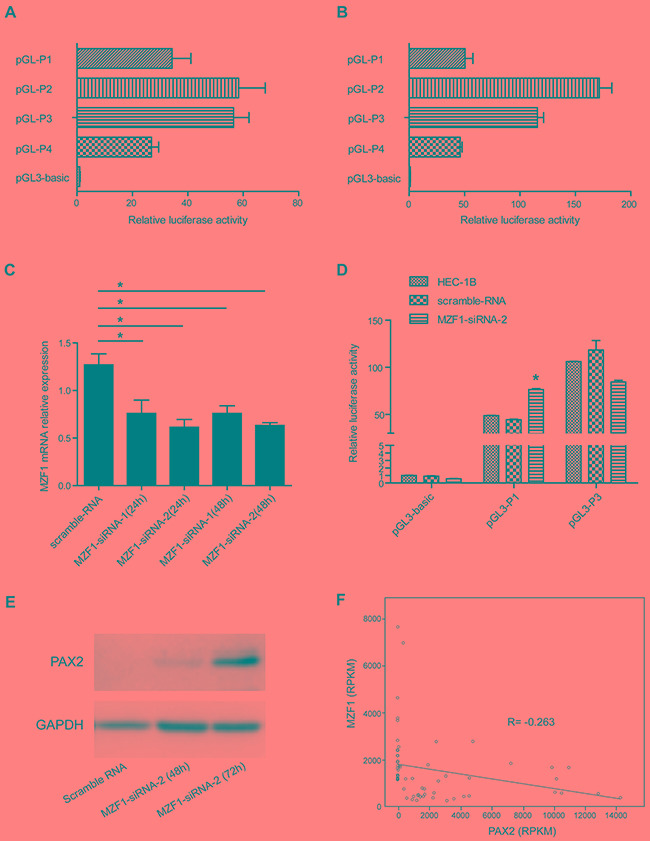
Deletion of P1(-683) to P3(-393) up-regulated the luciferase activity in both HEC-1A and HEC-1B cells, and knocking down MZF1 up-regulated PAX2 transcription and expression in cells containing the repressive transcriptional region of the PAX2 promoter **A.** Deletion analysis of the PAX2 promoter in HEC-1A cells. **B.** Deletion analysis of the PAX2 promoter in HEC-1B cells. **C.** MZF1 mRNA was down-regulated in HEC-1B cells 24 to 48 hours after transfection of MZF1-siRNA. **D.** Luciferase reporter gene activity was increased after knocking down MZF1 in HEC-1B cells transfected with pGL3-P1, but not up-regulated in cells transfected with pGL3-P3, which did not contain the repressive transcriptional region of PAX2 promoter (-683 bp to -393 bp). **E.** PAX2 protein was up-regulated in HEC-1B cells 72 hours after MZF1-siRNA transfection. F. By analyzing the RNAseq data from TCGA database, an negative correlation was demonstrated between the expression of MZF1 and PAX2 (R=-0.263, p=0.043). *p<0.05.

HEC-1A and HEC-1B cells were transfected with pGL3-P1, pGL3-P2, pGL3-P3 and pGL3-P4 vectors separately, and luciferase activity was assessed 24 hours after transfection. Deletion of -683 bp to -403 bp up-regulated the relative luciferase activity, indicating that this region is the repressive transcriptional region of the PAX2 promoter (Figure 5A and 5B), which is also corresponds to our M1 region that exhibits hypermethylation in the MassARRAY experiment.

### Transcription factor MZF1 down-regulates PAX2 expression by binding to repressive transcriptional region of PAX2 promoter

The transcription factor binding sites of the repressive transcriptional region of the PAX2 promoter (-683 bp to -393 bp) were predicted by using JASPAR database. MZF1 and NGFI-C/Egr-2 were predicted to bind to this region. The potential binding sites were GGGGA and GCCCA. We further focused on MZF1 because of its significant role in cancer.

Luciferase reporter gene activity was increased after knocking down MZF1 by siRNA in HEC-1B cells (Figure 5C) transfected with pGL3-P1; however, it was not up-regulated in HEC-1B cells transfected with pGL3-P3 that did not contain the repressive transcriptional region of the PAX2 promoter (-683 bp to -393 bp) (Figure 5D). The total cell lysate was collected, and the PAX2 levels were detected 48 and 72 hours after knocking down MZF1 in HEC-1B cells. The PAX2 level was up-regulated 72 hours after MZF1-siRNA transfection (Figure 5E). These results indicated that MZF1 may down-regulate PAX2 by binding to the repressive transcriptional region of the PAX2 promoter (-683 bp to -393 bp).

We further performed correlation analysis between the expression of MZF1 and PAX2 by analyzing the RNAseq data from the TCGA database. Both parametric and nonparametric analyses indicated a negative correlation between the genes (R=-0.263, p=0.043) (Figure 5F). These data support our observation that knocking down the expression of MZF1 up-regulated the expression of PAX2.

## DISCUSSION

PAX2 is expressed in multiple tumors, and its expression is essential for tumor cell survival in a variety of cancers, including cancers of the female reproductive tract. This study investigated the role and defined the promoter region that regulated the expression of PAX2 through DNA methylation and upstream transcription factors in endometrial cancer. We found that PAX2 was involved in endometrial cancer by stimulating cell growth and promoting cell motility. PAX2 overexpression in endometrial cancer was regulated by promoter hypermethylation via the transcription factor MZF1.

Conflicts exist in the studies of PAX2 in endometrial cancer. Some articles suggest that PAX2 is an oncogene in the development of endometrial cancer [19-22], but others consider it to be a tumor suppressor gene [23-28]. This contradictory result indicates that PAX2 may play a complex role in the development of endometrial cancer and may be due to the different genetic backgrounds among studies, differences in the antibody binding domains or insufficient sample sizes. Our results indicate that PAX2 is overexpressed in endometrial cancer compared with hyperplasia and normal endometrium, but there was no difference between hyperplasia and normal tissues. These results suggest that PAX2 may not be an important factor in the initiation of endometrial cancer but may be important in tumor progression. We further investigated the function of PAX2 and showed that it acted as a tumor promoter by promoting cell proliferation, migration and invasion in endometrial cancer cell lines. It is well accepted that there are two types of DNA methylation abnormalities. One type is genome instability caused by hypomethylation, such as chromosome instability and expression of oncogenes; the other is the silencing of tumor suppressor genes caused by the hypermethylation of CpG islands. The common rules are that hypomethylation induces activation, whereas hypermethylation induces suppression.

In this study, however, we identified a special regulation pattern of DNA methylation in PAX2 that differed from the common rules. In gynecological diseases, PAX2 is hypomethylated in tamoxifen-induced endometrial cancer [19]. We also performed bisulfite sequencing on the same region in this paper but revealed hypermethylation in endometrial cells (data not shown). We further quantified the methylation level of the upstream 648-bp fragment of the PAX2 promoter in 99 endometrial samples using MassARRAY system. The fragment contains 41 CpG sites, and a higher methylation level was observed in 14 sites in endometrial cancer samples compared with normal controls (p<0.05, the maximum interval is 23.8%). Of these sites, 7 consecutive CpG sites focused on the 5’ of M1 fragment (approximately 166 bp). These results are consistent with the bisulfite sequencing results in cell lines, indicating that the PAX2 promoter is hypermethylated in endometrial cancer. However, in contrast to the general rules of DNA methylation, we found the opposite result that PAX2 mRNA and protein levels were up-regulated in endometrial cancer.

Gene activation by promoter hypermethylation has been reported in recent years. Nabilsi [25] reported that the DNA methylation of survivin inhibited the binding of p53 to the gene, which led to the inhibition of p53-mediated survivin repression. It was also reported that 2% of the genome in prostate cancer was activated by hypermethylation. It was further verified that gene transcription was turned on if the hypermethylated sites were close to the transcription starting site (TSS) [26].

We further down-regulated the methylation level of the PAX2 promoter using a demethylating agent and verified that the decrease in the DNA methylation level down-regulated PAX2 mRNA and protein expression. Other PAX2 expression regulatory mechanisms may be present because down-regulating the methylation level did not completely inhibit its expression. In HEC-1A cells, PAX2 expression was slightly down-regulated, whereas the methylation level was significantly decreased, which indicated that DNA methylation was not the exclusive mechanism regulating PAX2 expression in this cell line. In Ishikawa cells, PAX2 expression was down-regulated by 5-Aza-CdR, however, PAX2 was initially hypomethylated, indicating that 5-Aza-dR might regulate PAX2 expression through other mechanism.

PAX2 was related to the development of multiple tumors; however, its regulatory mechanism was rarely reported. We identified a repressive regulatory region of the PAX2 promoter in endometrial cancer cells by cloning the 5’ flanking region. From the analysis of potential transcription factor binding sites in the upstream regulatory region of PAX2, we found that MZF1 and NGFI-C/Egr2 may be involved in the regulation of PAX2 transcription. MZF1 acts as an oncogene in the development of cervical cancer [27], colon cancer [28], breast cancer [29] and lung cancer [30] or a tumor suppressor gene in hematopoietic tumors [31] and ovarian cancer [32]. Early growth reaction gene (EGR) is a series of transcription factors that are induced by stimulations, such as hypoxia or infection. NGFI-C/Egr2 plays a significant role in the differentiation of T cells, the development of mouse brain and the myelination of peripheral nerves [33, 34]. According to the function of MZF1 and NGFI-C/Egr2, we focused on MZF1 for further study.

To verify the regulatory function of MZF1, we found an attenuation of repressive transcriptional activity at -683 to -393 bp in the PAX2 promoter after MZF1 was down-regulated. Furthermore, PAX2 was up-regulated at the protein level after knocking down MZF1 by siRNA. A negative correlation was also noted between these genes through a statistical analysis of the RNAseq data from the TCGA database. Thus, our data strongly suggest that MZF1 is involved in the regulation of PAX2 transcription by binding to the above region. The methylation level of the CpG site adjacent to the MZF1 binding site was 13.8% in endometrial cancer tissues and 4.0% in normal tissues, indicating that hypermethylation may attenuate the repressive regulation of MZF1 by preventing its binding to the PAX2 promoter and further up-regulate PAX2 transcription. However, the direct binding of MZF1 to the PAX2 promoter must be validated by further tests, such as ChIP, EMSA, and mutation of the MZF1 binding site or MZF1 protein domains.

## MATERIALS AND METHODS

### Cell lines

Human endometrial cancer cell lines HEC-1A, Ishikawa, RL95-2 and HEC-1B were obtained from the American Type Culture Collection (ATCC, Manassas, VA, USA) and maintained in McCoy's 5A (HEC-1A and Ishikawa) or DMEM-F12 (RL95-2 and HEC-1B) medium (Jinuo Co., Ltd, Shanghai, China) supplemented with 10% fetal bovine serum (Gibco-BRL, Rockville, IN, USA), 100 U/ml penicillin (Beyotime, Shanghai, China) and 100 μg/ml streptomycin (Beyotime, Shanghai, China). The cells were incubated at 37°C in 5% CO_2_.

### Primary endometrial cell culture and tissue samples

After approval from the institutional review board of Obstetrics and Gynecology Hospital of Fudan University, three normal endometrial epithelial cells (nEECs) of primary culture were obtained from biopsies under sterile conditions. Epithelial cell isolation was performed according to published procedures [35].

In total, 111 endometrial tissues were obtained from patients who underwent surgery from August 2008 to December 2012. We performed H&E staining in all samples to confirm that the biopsy had identical pathology of final diagnosis. All patients provided written informed consent permitting the use of their tissue for research at the time of specimen collection.

### Cell transfection

The PAX2 siRNA duplexes (siRNA-PAX2-1, 2) that target the sequences “5-CAUCAGAGCACAUCAAAUC-3” were synthesized by GenePharma Company (Shanghai, China). Full-length PAX2 cDNA (pCMV-Myc-PAX2) clone and vector (pCMV-Neo) were obtained from Origene (Rockville, MD, USA). Plasmids were amplified by Trans1-T1 Phage Resistant Chemically Competent Cells (TransGen Biotech, Beijing, China) with kanamycin selection and extracted from bacteria using HiSpeed Plasmid Midi and Maxi Kit for rapid transfection-grade purification (QIAGEN, Germany). HEC-1A and HEC-1B cells were seeded at 5×10^5^ cells/ml in 6-well plates, and RL95-2 were seeded at 1×10^6^ cells/ml. The following day, PAX2-siRNAs or the non-targeting siRNAs (100 nM) were added to media using Lipofectamine 2000 reagent (Invitrogen Inc., Carlsbad, CA, USA).

### Cell viability assay

Cell viability was evaluated by the cell counting kit-8 (CCK-8, Dojindo Molecular Technologies Inc., Gaithersburg, MD, USA). Cells were transfected with pCMV-Myc-PAX2, pCMV-Myc-Neo, siRNA-PAX2 or the non-targeting siRNA using Lipofectamine 2000 reagent. After a 6-hour incubation, cells were plated on 96-well plates at 5x10^3^ cells/well and incubated for 2, 3, 4 or 5 days at 37°C. After each indicated time, CCK-8 was aseptically added and incubated for 1 to 3 hours at 37°C. The absorbance was measured at 450 nm using a BioTeK Reader. Each experiment was conducted six times and repeated at least thrice.

### Cell migration and invasion assay

HEC-1A, HEC-1B and RL95-2 cells were transfected with PAX2 siRNA, non-targeting siRNA, pCMV-Myc-PAX2 or pCMV-Myc-Neo. After incubation for 72 hours, cell migration and invasion assays were performed according to published procedures [36]. Migrating cells were counted following a 24-hour incubation, and invading cells were counted following a 24 or 48-hour incubation using an Olympus light microscope in 5 randomly high power fields at x200.

### DNA isolation, bisulfite modification and bisulfite sequencing

Genomic DNA was extracted from primary cultured cells, cancer cell lines and frozen endometrial tissues using the QIAamp® DNA Mini and Blood Mini kit (Qiagen, Germany). Bisulfite reactions were performed using the EZ DNA Methylation-Gold™ kit (Zymo Research, CA, USA). Primer sequences for bisulfite sequencing of the PAX2 fragment B1 were 5’ TTGAATTAAGYGTTTTGGATTG 3’ (forward) and 5’ CAACCCTACCATCCACTAACC 3’ (reverse), which amplify the -764 to -447 region (16 CpG sites) upstream of PAX2. The annealing Tm was 55°C. The amplified PCR products were purified with Agarose Gel Extraction kit (Tiangen Biotech, Beijing, China) and ligated into the pEASY-T1 simple plasmid vector with a TA-cloning system (Transgene, Beijing, China). Plasmid-transformed *Escherichia coli* were cultured, and at least ten colonies were randomly chosen for plasmid extraction and sequenced with an ABI 377 automated sequencer using BigDye Terminator chemistry (Applied Biosystems) and the M13 primers.

### MassARRAY measurements of DNA methylation

The Sequenom MassARRAY platform (Institute of Biomedical Sciences, Fudan University, Shanghai, China) was used for the quantitative analysis of methylation in tissues. Briefly, the target DNA regions were amplified by PCR using bisulfite-modified DNA and specific primers. M1 region amplified -723 bp to -443 bp of PAX2 promoter, and the primer sequences were 5’- aggaagagagGTAAGGGGATTGGGGAGGTTTT -3’ (left) and 5’- cagtaatacgactcactatagggagaaggctACCCTACCATCCACTAACCAATAC -3’ (right). The M2 region amplified -468 bp to -71 bp of the promoter, and the primer sequences were 5’- aggaagagagGTATTGGTTAGTGGATGGTAGGGTT -3’ (left) and 5’- cagtaatacgactcactatagggagaaggctAACAAAAACAATAAATTCCACCACT -3’ (right). The PCR annealing Tm was 56°C, and sample preparation was performed according to “Training Instructions for EpiTYPER Quantitative Methylation Analysis Using MassCLEAVE for MassARRAY” (Sequenom). Further experimental analysis of the contents of DNA methylation was determined, as described previously [37].

### Treatment of endometrial cancer cells with a demethylating agent

Endometrial cancer cells were seeded in 6-cm culture dishes and incubated overnight. Cells were treated with 5 μM 5-aza-2’-deoxycytidine (Sigma, St Louis, MO, USA) for 72 hours for DNA and 96 hours for mRNA or protein extraction. All media were replenished daily, and all cells were harvested after 3 or 4 days of treatment.

### Immunohistochemistry and Immunocytochemistry

Immunohistochemistry (IHC), antigen retrieval and antibody dilution were optimized prior to the study onset (Mingrui Biotech, shanghai, China). To ensure uniformity, all sections were processed simultaneously. Immunohistochemistry was performed as described previously [38]. The slides were incubated with polyclonal antibodies against PAX2 (Invitrogen, CA, USA, 71-600, 1:50) at 4°C overnight. Semi-quantitative estimates were made using a score that evaluates the percentage of nuclear-positive cells. The percentage of positive cells was graded from 0 to 3 (0, negative; 1, <10 % positive cells; 2, 10–50 %; 3, 51–100 %). The scores were determined independently by two observers, and the average of the scores was used for evaluation.

For immunocytochemistry, cultured cells were fixed with 4% paraformaldehyde and treated with 1% Triton for 15 minutes. The following procedures were performed similar to IHC.

### cDNA synthesis and real-time PCR for PAX2

RNA was isolated from cells in culture using RNAprep Micro kit (Tiangen Biotech, Beijing, China). Reverse transcription was performed using RevertAid First Strand cDNA Synthesis kit (Fermentas, MA, USA). Quantitative RT-PCR was performed on Eco™ Real-Time PCR system using TaqMan® Gene Expression Assays (Hs99999903_m1 for β-actin, Hs01057417_ m1 for PAX2, Applied Biosystems, CA, USA) and KAPA PROBE FAST qPCR mastermix (KAPA Biosystems, Cape Town, South Africa). The thermal profile was 95°C for 10 minutes followed by 40 cycles of 95°C for 15 seconds and 60°C for 1 minute. PAX2 mRNA levels were calculated using the equation 2^-ΔΔCt^ and normalized to human β-actin mRNA levels.

### Western blotting

Cells were lysed in RIPA buffer. Equal amounts of protein were resolved by SDS-PAGE, transferred to PVDF membranes, and incubated with appropriate primary antibodies (PAX2 antibody, Invitrogen, CA, USA, 71-600, 1:200; GAPDH antibody, Epitomics, CA, USA, 5632-1, 1:5000). Immune complexes were detected with HRP-conjugated second antibodies (MT-bio, shanghai, China, 1:5000) and ECL chemiluminescence reagent (Thermo, MA, USA).

### Amplification and sub-cloning of 5’ flanking region of the PAX2 gene

Primers were designed according to the sequence of GenBank (NCBI Reference Sequence: NG_008680.1). The sequences of primers were shown as follows. Upstream of primers: P1: 5’-CCGCTCGAGGAGGGGACAAGACAAACTGC-3’; P2: 5’- CCGCTCGAGGAAGGAACGGAAGGGAACTC-3’; P3: 5’-CCGCTCGAG GCCCTCTGTCGTTACCTGAA-3’; P4: 5’-CCGCTCGAG CTCCGGCCGAGTCTTCTC-3’; downstream primer of all fragments: 5’-CCCAAGCTTTTGGCAGAGAAGTAGCAATCC-3’. The upstream primers all contain Xho I site and downstream primer contains a Hind III site at the 5’ end. The PCR program was conducted according to the touchdown method. The PCR products were P1 (-683 bp to +211 bp), P2 (-553 bp to +211 bp), P3 (-393 bp to +211 bp), and P4 (+10 bp to +211 bp).

### Construction of the recombined plasmids

The purified PCR products (P1, P2, P3 and P4) and pGL3-basic plasmid were excised with Xho I (New England Biolabs, Cat. # R0146L) and Hind III (New England Biolabs, Cat. # R3104L), dephosphorylated by dephosphorylation kit (New England Biolabs, Cat.# M0289S) and ligated into pGL3-basic vector (Promega, Madison WI, USA) using T4 DNA ligase (New England Biolabs, Cat.#M0202) to form the combined products: pGL3-P1, pGL3-P2, pGL3-P3 and pGL3-P4. The recombined plasmids were confirmed by sequencing using the general primers RVP3: CTAGCAAAATAGGCTGTCCC and GLP2: CTTTATGTTTTTGGCGTCTTCCA.

### Transient transfection of recombined plasmids

Recombined plasmids were amplified and purified by QIAGEN Plasmid Midi Kit (Qiagen, Germany). HEC-1A or HEC-1B was transfected with recombined plasmid (pGL3-P1/P2/P3/P4) and pRL-TK plasmid according to the manufacturer's instructions of for the Attractene transfection reagent (Qiagen, Germany). After culture for 24 hours, all samples were subject to the dual-luciferase reporter assay.

### Dual-luciferase reporter assay

Dual-luciferase reporter assay was conducted following the dual-luciferase reporter assay protocol (Promega, WI, USA). HEC-1B cells were transfected with scramble RNA or MZF1-siRNA followed by transfection with pGL3-P3, pGL3-P1, or pGL3-basic with the pRL-TK plasmid after 24 hours. Cells were cultured for an additional 24 hours, and luciferase activity was detected.

### Prediction of transcription factor binding sites in PAX2 promoter using JASPAR database

The repressive transcriptional region of PAX2 promoter was subjected to transcription factor binding site analysis using the web-based software JASPAR (http://jaspar.genereg.net/cgi-bin/jaspar_db.pl) as described previously [39]. Briefly, -683 bp to -393 bp of the 5’ upstream promoter of PAX2 was analyzed, and the threshold of percent-score was set above 80.

### Knocking down MZF1 by siRNA

After demethylating HEC-1B cells with 5-Aza-CdR (5 umol/L) for 72 hours, the cells were transfected with MZF1-siRNA-1, MZF1-siRNA-2, or scramble-siRNA (Shanghai Genepharma Co., Ltd synthesis) using the Lipofectamine 2000 reagent. The shRNA sequences are MZF1-siRNA-1, 5’-CCAGACACCAAGCUCAUUTT-3’; MZF1-siRNA-2, 5’-GAGGCUGGCGAUUACAUAATT-3’; and scramble-siRNA, 5’-UUCUCCGAACGUGUCACGUTT-3’. Whole cell lysate was collected 48 and 72 hours after transfection for detection of PAX2 expression.

### Statistical analysis

Statistical analysis was performed using SPSS 15.0 software (SPSS, Chicago, IL, USA). Nonparametric tests were utilized to analyze the immunohistochemistry, MassARRAY, the correlation between methylation and IHC scores, real-time PCR results and immuno cytochemistry. Unpaired Student's t-tests were used to analyze cell viability, migration and invasion assay. Both parametric and nonparametric test were used to analyze the correlation between the expression of MZF1 and PAX2. p-values < 0.05 were considered statistically significant.
